# Timing matters: diurnal spine length variation in pediatric patients during radiotherapy

**DOI:** 10.1186/s13014-024-02548-w

**Published:** 2024-11-20

**Authors:** Karin M. Meijer, Irma W.E.M. van Dijk, Tamara J. Schonk, Brian V. Balgobind, Anna Loginova, Niek van Wieringen, Arjan Bel

**Affiliations:** 1 Department of Radiation Oncology, Cancer Center Amsterdam, Amsterdam UMC, Amsterdam University Medical Center, University of Amsterdam, Amsterdam, The Netherlands; 2 Dmitry Rogachev National Medical Research Center of Pediatric Hematology, Oncology and Immunology, Moscow, Russia

**Keywords:** Pediatric cancer, Radiotherapy, Diurnal spine length variation, Geometrical uncertainties

## Abstract

**Background:**

During the day-night cycle, gravity and applied stress to the body mass and spine causes a decrease in body height, which is restored overnight. This diurnal spine length variation has not yet been quantified during radiotherapy. Therefore, we aimed to quantify diurnal spine length variation on cone beam CTs (CBCTs) of pediatric patients (< 18 years) who underwent radiotherapy.

**Methods:**

For this retrospective study, we included 32 patients (mean age 10.0, range 2.7–16.1 years) who received image guided radiotherapy between 2012 and 2018 in two institutes. Patients were included when they had two fractions per day, or when fractions were scheduled on varying time slots over the course of treatment. Daily CBCTs were registered to the planning CTs using two automatic registrations relative to the bony anatomy; one to vertebra T11 and one to vertebra L4. For each CBCT, the differences between the cranial-caudal (CC) position of the T11 and L4 vertebrae were calculated. To determine the diurnal spine length variation, the difference in vertebrae position between the morning and afternoon CBCTs was calculated. Furthermore, we investigated the possible correlation of diurnal spine length variation with the time slot differences (time interval) between CBCTs (Spearman’s ρ).

**Results:**

Overall, the median spine length variation was -1.0 (range -3.9–0.1) mm, and we found a significant reduction in spine length over the day (*p* < 0.001) with substantial variations between patients. Time intervals between CBCTs ranging from 4.0 to 9.5 h were not correlated with spine length reduction (ρ=-0.01; *p* = 0.95).

**Conclusions:**

We found a small but significant reduction in spine length (vertebrae T11 to L4) over the course of day in pediatric patients undergoing radiotherapy, measured on CBCT imaging. Spine length reduction did not correlate with CBCT time intervals. However, our results indicate that diurnal spine length reduction could induce a setup error during treatment, and therefore should be considered in pediatric radiotherapy.

## Background

During the day-night cycle, the height of the human body changes. The spine length decreases during the day due to fluid loss of the intervertebral discs, caused by gravitational and activity loading, and applied stress to the body mass [1–3]. When lying down, mostly during the night, pressure on the spine is released and fluid is absorbed back into the intervertebral discs due to osmotic pressure, and the spine length is restored again [4]. This change over day and night is called diurnal spine length variation. The spine length has been investigated in the field of ergonomics and clinical biomechanics in relation to work activities [5–7], and conditions such as chronic low back pain [8]. However, few studies used medical imaging to measure diurnal spine length variation [9, 10]. For radiation therapy, the impact of diurnal spine length variation has not been studied. The cone beam CT (CBCT), nowadays commonly used for daily patient position verification, provides images to quantify this variation.

Over the last decades, image guided radiotherapy (IGRT) using daily CBCT has become a standard treatment in pediatric radiotherapy to verify patient positioning during each fraction, and deliver the dose to the target according to the treatment plan and minimal dose to healthy tissues [11]. The ongoing developments of cancer treatment, including radiotherapy techniques, have led to an increase in survival rate [12]. Especially for children, higher survival rates also increase the risk of developing treatment-related late adverse effects, of which radiotherapy is a significant contributing factor [13]. This underlines the need for highly accurate treatment planning and dose delivery to target volumes. However, this is challenged by patient setup variations, interfractional (e.g. anatomical day-to-day variations) and intrafractional (e.g. breathing and peristaltic motion) variations, and delineation variability [14]. These geometrical uncertainties are mitigated by using safety margins, expanding the clinical target volume (CTV) to the planning target volume (PTV). Previous studies have quantified and evaluated inter- and intrafractional organ motion and reported large variations in motion, especially in cranial-caudal direction (CC), which could lead to differences in calculating safety margin sizes [15–19]. However, diurnal spine length variation as interfractional geometrical uncertainty for radiation treatment delivery has not yet been quantified. Previous quantifications of diurnal spine length variations in the field of biomechanics indicated a stature variation up to 17 mm over 24 h in adults [8]. Diurnal spine length variations could lead to an uncertainty if the pre-treatment planning CT is scheduled at a different moment during the day than the daily irradiation fractions and imaging (CBCT). Twice-daily fractions regimens for craniospinal irradiation (CSI) are more frequently applied for children [20, 21], and a variation in spine length could cause systematic errors in patient setup between the two fractions. Furthermore, when using a multi-isocenter approach for photon or proton CSI to encompass the full length of the spine, the distance between multiple (spinal) isocenters could be different due to spine length variations causing an uncertainty during patient setup [22, 23]. Hence, the possible effect of diurnal spine length variation on the definition of safety margins and radiation dose delivery has not been evaluated. Therefore, the aim of this study was to quantify diurnal spine length variation on CBCTs of pediatric patients who underwent radiotherapy.

## Methods

### Patient cohort

For this retrospective international cohort study, data of 32 patients were included from a total of 79 eligible pediatric (< 18 years) cancer patients who received radiotherapy with daily CBCT imaging, including flanks (for e.g. Wilms’ tumor or neuroblastoma), CSI and multi-isocenter total body irradiation (TBI) between 2012 and 2018 in two institutes. Patients were selected when they underwent a twice-daily hyperfractionated regimen (*N* = 14), or when consecutive daily fractions were scheduled on varying time slots during the day (*N* = 18). Patients were excluded from diurnal spine length analysis when the time slot differences between acquisition of two CBCT scans were not long enough (<4 h; *N* = 27), or if the lower part of the thoracic spine and the entire lumbar spine were not visible on imaging (*N* = 20). Patient characteristics are described in Table 1. All patients were treated in supine position, and for 14 (44%) patients a vacuum mattress for immobilization was used. Furthermore, we collected information on the daily activity of patients during the period of treatment. Seven patients were normally active during the day, and two patients showed less activity due to illness and fatigue during treatment. Furthermore, three patients were wheelchair-bound, and three patients were passive/bedridden during the day. For 17 patients, information on daily activity was not reported.

**Table 1:** Characteristics of the 32 pediatric patients treated between 2012 and 2018 at the Amsterdam UMC (*N* = 18) and DR NMRC-PHOI Moscow (*N* = 14)

	Mean (SD; range)	N	(%)
Gender^a^			
Male		26	(81.3)
Female		6	(18.8)
Age at first RT fraction (years)	10.0 (3.8; 2.7–16.1)		
Height (cm)	140.7 (22.6; 90.0–182.0)		
Weight (kg)	33.3 (14.8; 12.0–68.0)		
Type of primary cancer^a^			
Leukemia^b^		12	(37.5)
Brain/CNS tumors			
Medulloblastoma		7	(21.9)
Ependymoma		2	(6.3)
Neuroblastoma		1	(3.1)
Other^c^		4	(12.5)
Rhabdomyosarcoma		3	(9.4)
Lymphoma		2	(6.3)
DSRCT		1	(3.1)
Treatment site			
Craniospinal		13	(40.6)
Abdominal (incl. flank)		5	(15.6)
TBI		14	(43.8)

^a^Percentages do not add up to 100 due to rounding, ^b^Including: acute lymphoblastic leukemia (*N* = 10), acute myeoloid leukemia (*N* = 1), acute bilineal leukemia (*N* = 1): ^c^Including: germinoma (*N* = 2), atypical teratoid rhabdoid (*N* = 1), glioma (*N* = 1)

*Abbreviations* *RT*, radiotherapy; *SD*, standard deviation; *CNS*, central nervous system; *DSRCT*, desmoplastic small round cell tumor; *TBI*, total body irradiation; *UMC*, University Medical Center; *DR NMRC-PHOI*, Dmitry Rogachev National Medical Research Center of Pediatric Oncology, Hematology and Immunology

### Treatment and imaging data

Pre-treatment planning CT scans were acquired according to institution based standard protocols, which were used as the reference image (refCT) for the spine length measurements. For each patient, one thorax-abdomen refCT was used (N_CT_=32; slice thickness 2.5–7.5 mm). Radiation treatment was delivered using photon linear accelerators with an integrated CBCT scanner (Synergy, Elekta oncology Systems, Crawley, UK). Since no CBCT imaging visualizing the full spine length was available, we used abdominal CBCTs on which the lower thoracic spine and lumbar spine were visible. A total number of 142 CBCTs (N_CBCT_; range 2–6 per patient) were used to quantify diurnal spine length variation (Table 2). CBCT energy ranged from 100 to 120 kV acquired with a tube current of 10 to 40 mA. The gantry rotation varied between 195 and 360 degrees, with a range of 174 to 707 projection images per CBCT and a slice thickness of 1 mm.

**Table 2:** Number of abdominal CBCTs (total 142) per patient used for diurnal spine length quantification in 32 pediatric patients (< 18 years) treated with radiotherapy

Number of CBCTs	N	(%)^a^	Number of CBCT pairs
2	8	(25.0)	8
3	2	(6.3)	4
4	6	(18.8)	12
6	16	(50.0)	48

^a^Percentages do not exactly add up to 100 due to rounding

*Abbreviations* *CBCTs*, cone beam computed tomography

For each patient, the CBCTs acquired in the morning (CBCT_1_) were paired to the CBCTs acquired in the afternoon (CBCT_2_). The number of CBCT pairs ranged from one to three per patient, and this resulted in 72 pairs (data points) to quantify diurnal spine length variation (Table 2). Additionally, for each pair the time slot difference between CBCT_1_ and CBCT_2_ was calculated based on the time of day of image acquisition. We refer to this as the time interval between imaging. For analysis, the CBCT time interval was assumed to be representative for time intervals between planning CTs and CBCTs.

### Diurnal spine length variation

**Fig. 1:**
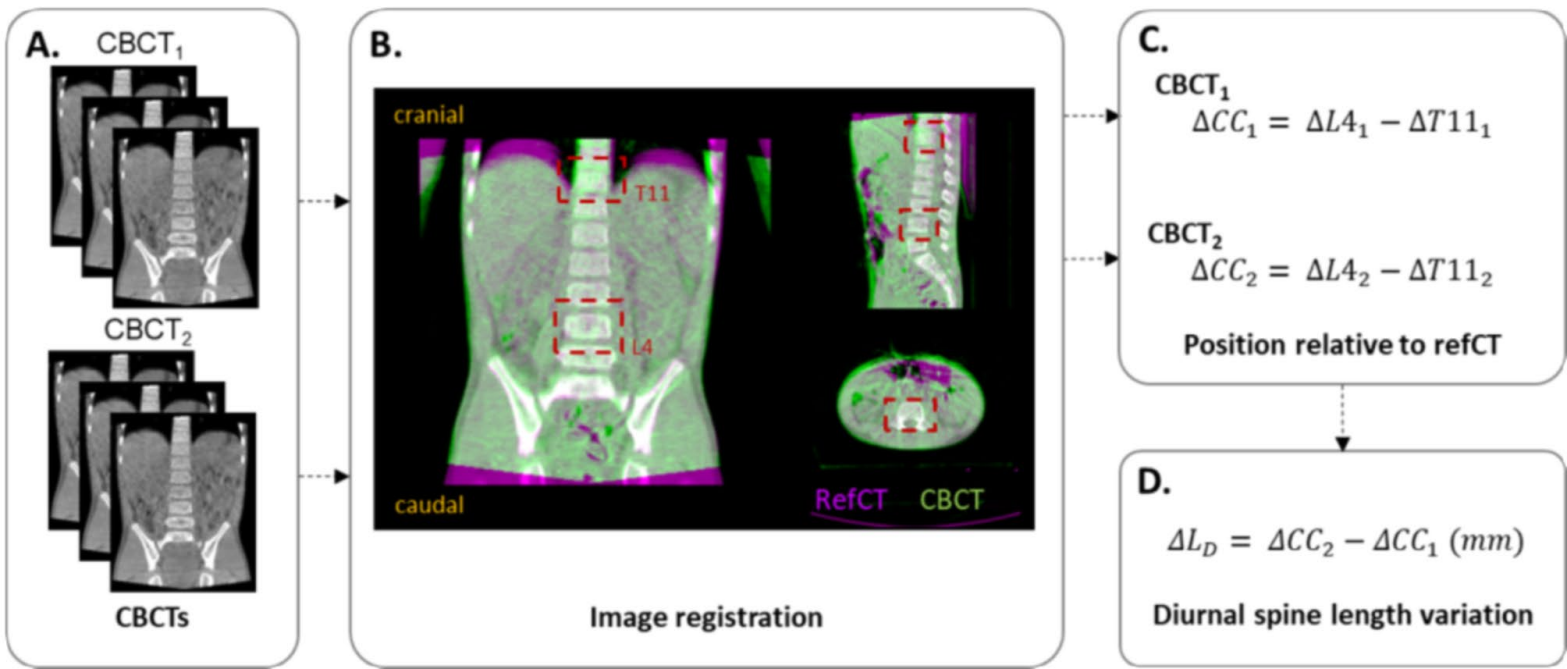
**A**) A total of 142 cone beam CTs (CBCTs) acquired in the morning (CBCT_1_) and the afternoon (CBCT_2_) were collected, resulting in 72 CBCT pairs. **B)** Each CBCT was registered to the reference CT (refCT) relative to the bony anatomy using defined regions of interest (ROIs) of vertebrae T11 and L4. **C)** Per CBCT, the position of the vertebrae in cranial-caudal (CC) direction were extracted, **D)** and the difference in CC position between CBCT_2_ and CBCT_1_ was used to calculate diurnal spine length variation (ΔL_D_) in mm

To quantify the diurnal spine length variation, we used automated rigid translational registrations using Elekta X-ray Volume Imaging (XVI) software (version 5.0; Elekta Oncology Systems). First, two regions of interest (ROIs) were defined, one including vertebra T11 and one including vertebra L4. Per patient, all CBCT images were registered to the same refCT, relative to the bony anatomy of the two vertebrae, based on the defined ROIs using an automatic chamfer match algorithm (Fig. 1.A-B). It was visually inspected if the cranial border of T11 and the caudal border of L4 were properly registered and, if necessary, manual corrections were performed. For both CBCT_1_ and CBCT_2_, the position of vertebrae T11 and L4 in CC direction (mm), relative to the refCT, was extracted (Fig. 1.C). To calculate the diurnal spine length variation, the positions of both vertebrae T11 and L4 on CBCT_1_ were compared to those on CBCT_2_. Hence, the diurnal spine length variation (ΔL_D_) was defined as the difference in positions of the vertebrae in CC direction between CBCT_2_ (ΔCC_2_) and CBCT_1_ (ΔCC_1_) (Fig. 1.D).

### Statistical analysis

For all CBCT pairs, we calculated the overall median and range of the diurnal spine length variation. We used the Shapiro-Wilks test to check the data for normal distribution. Since not all data fitted the normal distribution, non-parametric tests were used for analyses. With the null hypothesis being that there is no spine length variation (0 mm), we used the sign test to test the spine length variations for significance. Furthermore, we investigated possible correlations of diurnal spine length variation with the time interval (in hours) between CBCT_2_ and CBCT_1_, and with patients’ height and weight, using linear regression analyses (Spearman’s correlation coefficient; ρ). For all statistical tests, a *p* value of < 0.05 was considered significant. All statistical analyses were performed using the software package RStudio [24].

## Results

**Fig. 2:**
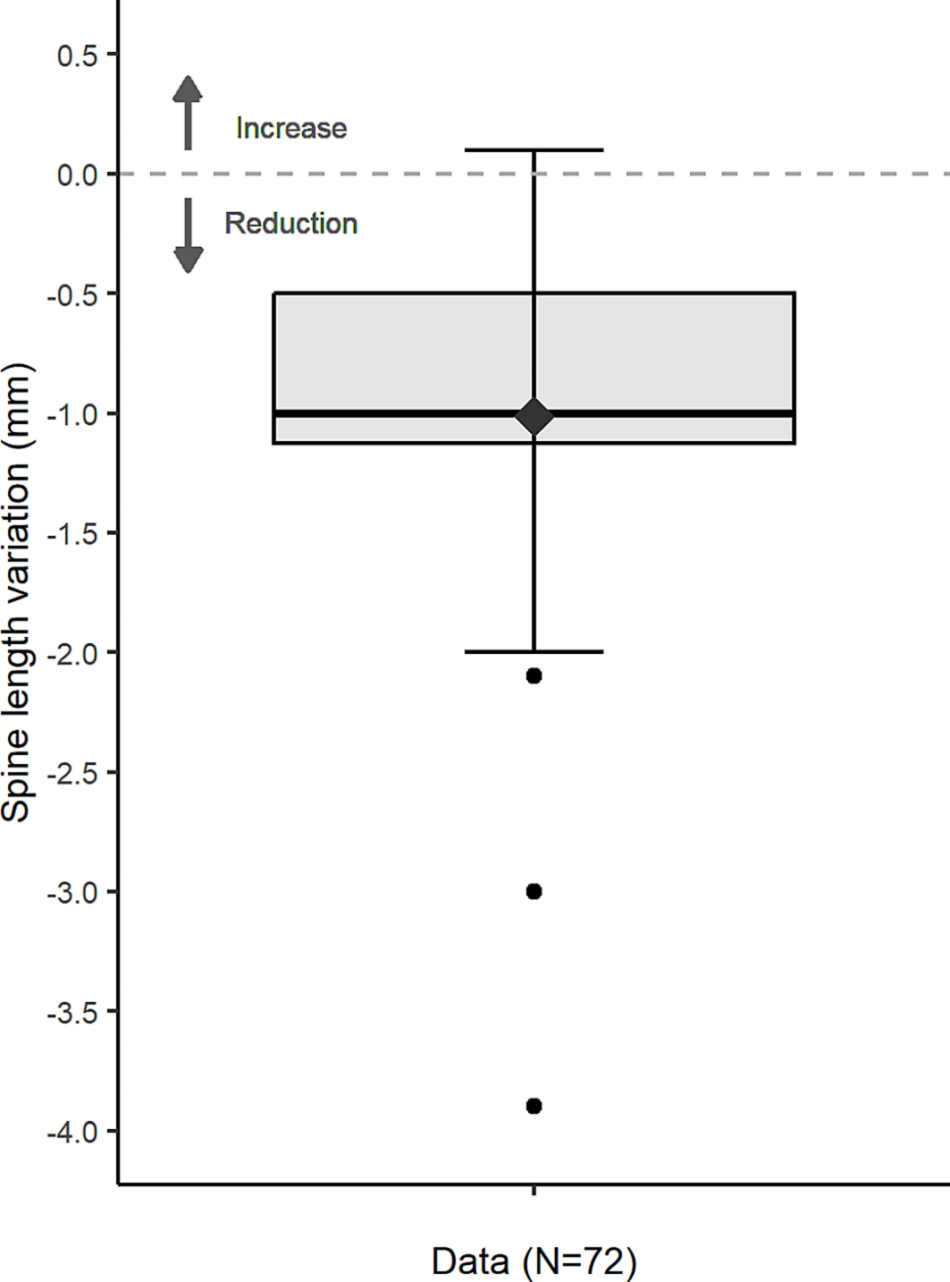
Boxplot showing the variation in spine length ΔL_D_ for vertebrae T11 to L4, based on 72 CBCT pairs. Negative values indicate a reduction of the spine length. The diamond represents the mean. The horizontal bar, box and whiskers represent the median, 50th percentile (inter quartile range; IQR), and the highest and lowest value within 1.5xIQR, respectively

For each calculation of diurnal spine length variation, two automated registrations of both T11 and L4 were performed of the CBCTs with the refCT, totaling *N* = 288 registrations. After visual inspection 16 (5.6%) registrations needed to be manually corrected. The overall median spine length variation was -1.0 mm with a range of -3.9 to 0.1 mm (Fig. 2). The sign test showed that the spine length variation was significantly smaller than the reference value 0 (*p* < 0.001), meaning there was a significant reduction of the spine length between CBCT_2_ and CBCT_1_.

For one CBCT pair we found a positive value of 0.1 mm spine length variation, indicating a small increase of the spine length, and in four patients no difference in spine length was found. However, for the majority of the data points (67/72; 93.1%) we found a reduction of the spine length. As spine length variation is caused by gravitational and activity loading, we compared the results of patients with normal daily activity (*N* = 7) and patients who were bedridden (*N* = 3). The median spine length variation of patients with normal activity (*N* = 14 CBCT pairs) was -0.6 mm, with a range of -2.0–0.0 mm. For bedridden patients (*N* = 7 CBCT pairs), we found a median spine length variation of -1.0 mm, with a smaller range of -1.0–0.0 mm.

The mean time interval between the refCT and CBCTs was 3.2 ± 2.2 (range 0.1–10.6) hours, and 5.5 ± 1.1 (range 4.0–9.5) hours between CBCT_1_ and CBCT_2_. We found no correlation between diurnal spine length variation and the time interval between imaging (ρ=-0.01; *p* = 0.95). As children aged between 2.7 and 16.1 years were included, patients’ height and weight largely varied (Table 1). For both height and weight, the Spearman’s ρ coefficient showed a negative, but weak (ρ=-0.18 and ρ=-0.14, respectively) and not significant (*p* = 0.31 and *p* = 0.46, respectively) correlation with diurnal spine length variation.

## Discussion

In this retrospective study, we investigated diurnal spine length variation in pediatric patients undergoing radiotherapy. We found a significant reduction of spine length measured from vertebrae T11 to L4 on CBCTs acquired in the morning compared to the afternoon. Furthermore, we found no correlations of diurnal spine length variation with the time interval in hours between CBCT images, nor with patients’ height and weight. Data was collected from two institutes, resulting in the first multicenter cohort study to quantify diurnal spine length variations in pediatric patients using CBCT imaging acquired during the course of radiotherapy. Similar to our results, previous studies performed in different clinical fields found a decrease of patients’ height over the course of time during the day [2, 6–8, 25]. The most commonly used measuring apparatus was the standing or seated stadiometer, which measures patients’ height from head to toe or head to seat [6, 7]. Therefore, direct comparison of the diurnal spine length variation results is hampered as we quantified spinal shrinkage only partially.

During the day, especially the cervical to lumbar vertebrae (*N* = 24) endure gravitational and activity loading, with shrinkage of the intervertebral discs as a result [1, 26, 27]. In our study, measurement of the spine length variation was restricted to vertebrae T11 to L4, as this part of the spine was visible on all imaging. Therefore, our results are based on six vertebrae, and not the full spine length. Thus, when taken into account the entire spine, the absolute diurnal spine length variation would increase. Previous studies have investigated the relation between pressure on the lumbar spine and spinal shrinkage, but no general human model for all vertebrae is available to estimate the diurnal variation of all vertebrae from the cervical to lumbar spine [4, 26]. Based on our results and assuming that the pressure on the spine is equally distributed over all vertebrae, the diurnal spine length reduction could theoretically increase up to 15.6 mm, causing a large geometrical uncertainty. Even though this is likely an overestimation, a study including adult patients showed spine length reductions of similar magnitude [8]. However, the impact of an absolute spine length reduction is comparatively different for younger children (< 5 years) than for (almost) full grown children (e.g. 17 years) or adults. Therefore, a correction based on patients’ height should be applied. Still, diurnal spine length variations should be considered as a factor that could induce a setup error during radiotherapy.

We found a significant reduction of the spine length during the day, but with considerable variations between patients. Although in some cases the diurnal spine length variation results were small (< 0.2 mm), the variations up to 4.0 mm could contribute to setup errors which occur during radiation treatment. For photon and proton CSI, multiple spinal fields can be used to cover the entire length of the spine and an anatomical change of the spine length could cause a shift in the patients’ optimal alignment [28]. These shifts could cause a gap or overlap in spinal fields which would consequently lead to an under- or overdosage of the target and/or organs at risk [29, 30]. Furthermore, patients with leukemia are often treated with TBI in preparation of bone marrow or stem cell transplantation, and optimal dose coverage of multiple fields reduces the risk of treatment failure [30]. Therefore, such positioning errors during patient set-up need to be corrected for by using optimal field overlap regions [23, 31].

In most cases of IGRT, one pre-treatment CT is used for all delivered fractions. If this planning CT is acquired in the morning and daily fractions are scheduled in the afternoon, the spine length variation could systematically lead to inaccuracies when patients’ bony anatomy is used for image registration of daily CBCT to the planning CT for patient positioning [14]. Our results showed diurnal spine length variation over 1.0 mm. Even though variations were relatively small, in our field of high precision radiation treatment even these variation might cause insufficient dose coverage the target and/or organs at risk. However, in our study we were not able to quantify these possible consequences for treatment planning and dose delivery. These uncertainties caused by diurnal spine length variations could be reduced by preferably scheduling the radiation fractions in the same timeslots as the pre-treatment planning CT was acquired. However, in clinical practice this might be difficult to achieve. Furthermore, for patients who receive hyperfractionated radiotherapy with two-daily fractions [20, 21, 32], the spine length variations may still affect at least one fraction due to the time difference. In our study, time intervals between CBCT image acquisitions varied largely, ranging from 4.0 to 9.5 h, because of protocol differences between institutes and the small number of included patients treated with hyperfractionated regimens. A time trend of the diurnal spine length was not demonstrated in our results. To investigate the possible correlation between time interval and spine length variation, a more consistent time interval of e.g. 6 to 8 h for each patient would be needed, which is representative for two-daily fraction regimens [20, 32].

As 2D measurement methods (e.g. a software ruler) to quantify the distance between two vertebrae in CC direction do not take into account anatomical and geometrical variations of the vertebrae in other directions, we used a 3D automated image registration algorithm. The rotational components of the vertebrae, particularly the lumbar lordosis, were not taken into account in our analysis. The lumbar lordosis could have led to an uncertainty in measurements, and the quantification of the spine length variation could be improved by also taking into account the rotations of the vertebrae. However, as all patients were treated in the same (supine) position for each fraction, the possible effect of the lumbar lordosis on the analysis was considered to be minimal. In addition, 14 (44%) patients were fixated using a vacuum mattress to secure stable patient positioning, which contributed to the reproducibility of the setup. Imaging data was acquired based on institutes’ protocols, with different acquisition parameters for CTs and CBCTs, including different slice thicknesses. A larger slice thickness could have caused uncertainties in the calculation of spine length variations as our analysis was performed in CC direction only. Such uncertainties could have led to a statistical error, and possibly to the single result of a slight expansion (0.1 mm) of the spine during the day. However, since we used a 3D image registration method, and not a 2D measurement tool (e.g. on a sagittal reconstruction), the impact of larger slice thicknesses is expected to be minimal.

Furthermore, we only included one to three data points per patient because more CBCTs pairs with sufficient time intervals between the scans were not available. To reduce a possible measurement error, it would be recommended to have more repeated measurements per patient. Furthermore, to account for the individual variation of diurnal spinal reduction a patient-specific approach by using online adaptive radiotherapy can be considered in the treatment of pediatric patients [33].

Previous studies in the fields of ergonomics and clinical biomechanics reported on diurnal spine length variations and found differences in spinal shrinkage when patients endured different activity loading or gravitational loading [1, 6, 34]. Patients’ weight is a factor in the gravitational loading, however, our results showed no significant correlation between weight and diurnal spine length variation. Furthermore, the correlation between patients’ height and diurnal spine length variation showed similar results. Even though the correlations were negligible, we noticed a negative trend between height and spine length reduction, indicating more shrinkage in heavier and taller patients. In our analysis, the impact of activity loading on the spine could not be statistically analyzed because the reporting of daily patient activity was scarce. We were able to compare two small sample sizes, and our results showed no considerable differences in spine length variation between patients reported to be normally active during the course of treatment compared to bedridden patients. Previous studies have shown the impact of daily activity on diurnal spine length variation [1, 26]. However, with the limited number of patients in both samples, no further statistical analysis was feasible. Since patient activity was not reported for each day of image acquisition, we assumed that patient activity remained the same during the course of treatment. Therefore, no definitive conclusions can be drawn from our results and a larger patient cohort would be needed including data on patients’ daily activity. However, the variations indicate that spine length reduction should be taken into account as a factor that can induce a geometrical uncertainty during radiotherapy. Moreover, a better understanding of the possible consequences of diurnal spine length variation on dose delivery is essential to fully advance from the high precision and patient-specific treatment approaches.

## Conclusions

In this retrospective study, we found a small but significant diurnal spine length reduction in pediatric patients, measured for vertebrae T11 to L4 on CBCT imaging. Spine length reduction did not correlate with the time slot differences between CBCT scans, but our results implicate that diurnal spine length reduction could induce a setup error during treatment. Hence, diurnal spine length reduction is a factor to be considered in pediatric radiotherapy when fractions are scheduled at varying time slots over the course of treatment, or when two-daily fraction regimens are applied.

## Data Availability

No datasets were generated or analysed during the current study.

## Abbreviations

CBCT: Cone beam computed tomography
IGRT: Image guided radiotherapy
CTV: Clinical target volume
PTV: Planning target volume
CC: Cranial-caudal
CSI: Craniospinal irradiation
TBI: Total body irradiation
UMC: University Medical Centers
DR NMRC-PHOI: Dmitry Rogachev National Medical Research Center of Pediatric Oncology, Hematology and Immunology
RT: Radiotherapy
SD: Standard deviation
CNS: Central nervous system
DSRCT: Desmoplastic small round cell tumor
refCT: Reference computed tomography
XVI: X-ray Volume Imaging
ROI: Region of interest
